# High versus low measurement frequency during 24-h ambulatory blood pressure monitoring - a randomized crossover study

**DOI:** 10.1038/s41371-023-00868-0

**Published:** 2023-10-11

**Authors:** Martin B. Thomsen, Jakob Nyvad, Kent L. Christensen, Mark Reinhard, Niels Henrik Buus

**Affiliations:** 1 Department of Renal Medicine, Aarhus University Hospital, Aarhus, Denmark; 2 Department of Clinical Medicine, Health, Aarhus University, Aarhus, Denmark; 3 Department of Cardiology, Aarhus University Hospital, Aarhus, Denmark

**Keywords:** Diagnosis, Hypertension

## Abstract

Ambulatory blood pressure monitoring (ABPM) may be stressful and associated with discomfort, possibly influenced by the number of cuff inflations. We compared a low frequency (LF-ABPM) regimen with one cuff inflation per hour, with a high frequency (HF-ABPM) regimen performed according to current guidelines using three cuff-inflations per hour during daytime and two cuff-inflations during night time. In a crossover study, patients underwent ABPMs with both frequencies, in a randomized order, within an interval of a few days. Patients reported pain (visual analogue scale from 0 to 10) and sleep disturbances after each ABPM. The primary endpoint was the difference in mean 24 h systolic BP (SBP) between HF-ABPM and LF-ABPM. A total of 171 patients were randomized, and data from 131 (age 58 ± 14 years, 47% females, 24% normotensive, 53% mildly hypertensive, and 22% moderately-severely hypertensive) completing both ABPMs were included in the analysis. Mean SBP was 137.5 mmHg (95% CI, 134.8;140.2) for HF-ABPM and 138.2 mmHg (95%CI, 135.2;141.1) for LF-ABPM. The 95% limits of agreement were −15.3 mmHg and +14.0 mmHg. Mean 24 h SBP difference between HF-ABPM and LF-ABPM was −0.7 mmHg (95%CI, −2.0;0.6). Coefficients of variation were similar for LF-ABPM and HF-ABPM. Pain scores (median with interquartile range), for HF-ABPM and LF-ABPM were 1.5 (0.6;3.0) and 1.3 (0.6;2.9) during daytime, and 1.3 (0.4:3.4) and 0.9 (0.4;2.0) during nighttime (*P* < 0.05 for both differences). We conclude that LF-ABPM and HF-ABPM values are in good agreement without any clinically relevant differences in BP. Furthermore, LF-ABPM causes a relatively modest reduction in procedure-related pain.

## Introduction

Ambulatory blood pressure monitoring (ABPM) is recommended as the most accurate non-invasive method for the assessment of blood pressure (BP) and BP-related cardiovascular risk [1], especially when performed for 24 hours (h) with measurements during both wakefulness and sleep [2]. The 2021 European Society of Hypertension (ESH) practice guidelines recommend ABPM or home BP for all patients with suspected hypertension [1]. However, a major limitation of ABPM is patient reluctance to undergo more than one ABPM because of pain, discomfort, and reduced sleep-quality caused by repeated cuff inflations [1, 3–5].

The current ESH guidelines and the International Society of Hypertension recommend 2–3 cuff inflations every hour during ABPM [1]. Only little evidence exists supporting this recommendation, and current guidelines are mainly based on consensus. In fact, previous studies have suggested that average BP during ABPM using hourly measurements is similar to average BP using higher measurement frequencies [6–14]. However, these studies have generally focused on nighttime BP [6] or included only normotensive individuals [7]. Furthermore, most studies were based on ABPMs using the number of cuff inflations recommended by guidelines, and then afterwards mimicking a lower measurement frequency in the analysis by randomly removing measurements [8–15]. Consequently, these studies could not directly provide information on the effect of fewer cuff inflations regarding pain, discomfort, sleep quality, and BP itself [8–15]. The question of whether measurement frequency influences 24 h BP therefore remains unanswered.

Previous investigations suggest that BP transiently rises during cuff inflation, particularly in hypertensive patients [16–18]. This effect could be amplified by many repeated measurements causing a measurable reactive rise in BP over the course of a 24 h ABPM. In a recent non-randomized study, we found indications that ABPM may indeed be influenced by the measurement frequency, since more frequent cuff inflations resulted in significantly higher systolic BP (SBP) compared to hourly measurements. This finding was most pronounced in moderate to severely hypertensive patients [19].

In the present randomized crossover study, we compared ABPM using one measurement per hour for 24 h with current guideline recommendations of three measurements per hour during daytime and two per hour during nighttime.

## Methods

### Participants

Patients (≥ 18 years) were recruited from the Hypertension Clinic at Aarhus University Hospital, Denmark and from two local private cardiology clinics. Suspected treatment resistant hypertension was the main reason for attending these clinics. Exclusion criteria were pregnancy, change in antihypertensive medication within 14 days prior to inclusion, treating physician preference of using an ABPM-device other than the devices described below, arm circumference above or below available cuff sizes (20–42 cm), inability to give informed consent, or not expecting to sleep during the night on either day of ABPM (nightshift work or other reasons). Patients were not invited to participate if permanent- or persistent atrial fibrillation were noted in their electronic patient records three years prior to inclusion. Information about patient characteristics, comorbidities, and medication were obtained from electronic patient records.

Included patients were assigned a hypertension category based on their first ABPM. Normotension/controlled hypertension was defined as 24 h SBP/diastolic BP (DBP) < 130/80 mmHg, mild hypertension as 24 h SBP 130–149 mmHg and moderate/severe hypertension as 24 h SBP ≥ 150 mmHg. Chronic kidney disease was defined as estimated glomerular filtration rate below 60 ml/min/1.73 m^2^ on two consecutive blood samples more than three months apart or when urine albumin-creatinine ratio was above 30 mg/g in two out of three urine samples. Diabetes was defined as a hemoglobin A1c concentration above 48 mmol/mol or at least one prescribed antidiabetic drug. Ischemic heart disease was considered present in patients with previous coronary interventions, if significant coronary calcification had been documented on cardiac computerized tomography scan or coronary angiography or in the case of clear angina symptoms.

### Materials

Prior to the first ABPM, a Microlife watchBP Office BP device (Microlife, AG Widnau, Switzerland) was used for bilateral BP measurements. If the inter-arm difference was larger than 10/5 mmHg (SBP/DBP) the ABPM device was fitted to the arm with the highest BP. If not, patients were given the option to choose which arm the cuff was fitted on. The same arm was used for both ABPMs.

Spacelabs Ontrak and Spacelabs 90217A (Spacelabs Healthcare, Snoqualmie, Washington, USA) were used for the ABPMs. Both devices are validated according to internationally recognized standards [20]. Arm circumference was measured to select the correct cuff size. Sentinel software (v11.5.2.13260, Spacelabs Healthcare, Snoqualmie, Washington, USA) was used to retrieve data from the ABPM device. A recording was considered acceptable if 70% or more of the measurements were successful [21]. The software was preset to discard any SBP measurements outside of the range 70–240 mmHg, and any DBP outside the range 40–150 mmHg.

During high frequency ABPM (HF-ABPM), daytime was predefined as 07:00–23:00 and BP was measured every 20 min in this time interval and every 30 min for the remaining eight hours. Low frequency ABPM (LF-ABPM) measured BP once every hour during all 24 h. Patients reported individual bed- and rising times after each ABPM, and these were used to define awake and sleep measurements post hoc. If a planned measurement failed, the device was programmed to perform one extra measurement within two minutes. Initially, four measurements were made during fitting to ensure that the device worked correctly. These initial measurements and measurements performed after 24 h of recording, were removed from our analysis.

### Study protocol

All patients scheduled for a planned ABPM were screened and eligible patients were asked to participate. Patients who accepted to enter the study were randomized 1:1, using an online random number generator [22], to either HF-ABPM or LF-ABPM as their first recording.

Patients were prepared for the ABPM in accordance with current guidelines [1], and instructed to keep the two measuring days similar in terms of physical activity, bed/rising times, medication, and time of medication ingestion. We did not apply restrictions concerning caffeine or alcohol intake. The second ABPM was conducted using the alternative measurement frequency no earlier than 48 h after finishing the first ABPM. At the time of the second ABPM, neither the person fitting the cuff nor the participant, were aware of the results of the first measurement. To minimize inter-device variation, the exact same ABPM device and cuff was used in both ABPM for almost all patients. However, three patients were unable to return to the hospital for the second fitting and were therefore handed two devices, each configured to one of the frequencies. They were fitted with the first device and instructed how and when to fit the second device themselves at home.

Immediately after each ABPM, the patients filled in a questionnaire concerning antihypertensive medication taken for the last 24 h, self-perceived sleep interruptions attributed to the measurements, symptoms from the arm after removal of the cuff, and pain perceived during day and night measurements. Pain associated with cuff inflations was reported on a visual analogue scale (VAS) from 0 to 10. Only patients who completed both questionnaires were included in the questionnaire analysis.

### Statistical evaluation

The primary outcome was the difference in 24 h mean SBP between HF-ABPM and LF-ABPM. Secondary outcomes were mean differences in SBP for day- and nighttime, DBP values (24 h, daytime, and nighttime means), 95% limits of agreement (95% LoA) interval, coefficient of variation (CV) for both SBP and DBP values calculated as standard deviation (SD) / mean × 100, and questionnaire data as reported above. The mean difference and the LOA between HF- and LF-ABPM were calculated using Bland-Alman plots and the relationships between differences and means were tested with simple linear regression and plotted appropriately, 24 h means are also presented in scatter plots with a line of equality as reference [23]. Using a 2-sided significance level of 0.05, a power of 0.9, and a SD of 13.5 mmHg [19] we calculated a needed sample size of about 120 patients to detect a 4 mmHg SBP difference between LF-ABPM and HF-ABPM.

Data were analyzed using STATA (Version 17, StataCorp, College Station, TX, USA). Continuous variables were evaluated for normal distribution using QQ-plots and histograms. BP results are presented as means with 95% confidence intervals (CI). All other continuous, normally distributed variables are presented as means ± SD, and data with a skewed distribution are presented as median with interquartile range (IQR). Dichotomous variables are presented as number of patients with % of total population. For independent data unpaired t-test, Mann-Whitney-U test and chi-squared-test were used for normally distributed, skewed, and dichotomous data, respectively. For paired data a paired t-test, Wilcoxon matched-pairs signed-rank test, and McNemar’s test were used for normally distributed, skewed, and dichotomous, data respectively. A *P*-value less than 0.05 was considered statistically significant. The main author has full access to all the data in the study and takes responsibility for its integrity and the data analysis.

## Results

### General characteristics

**Fig. 1:**
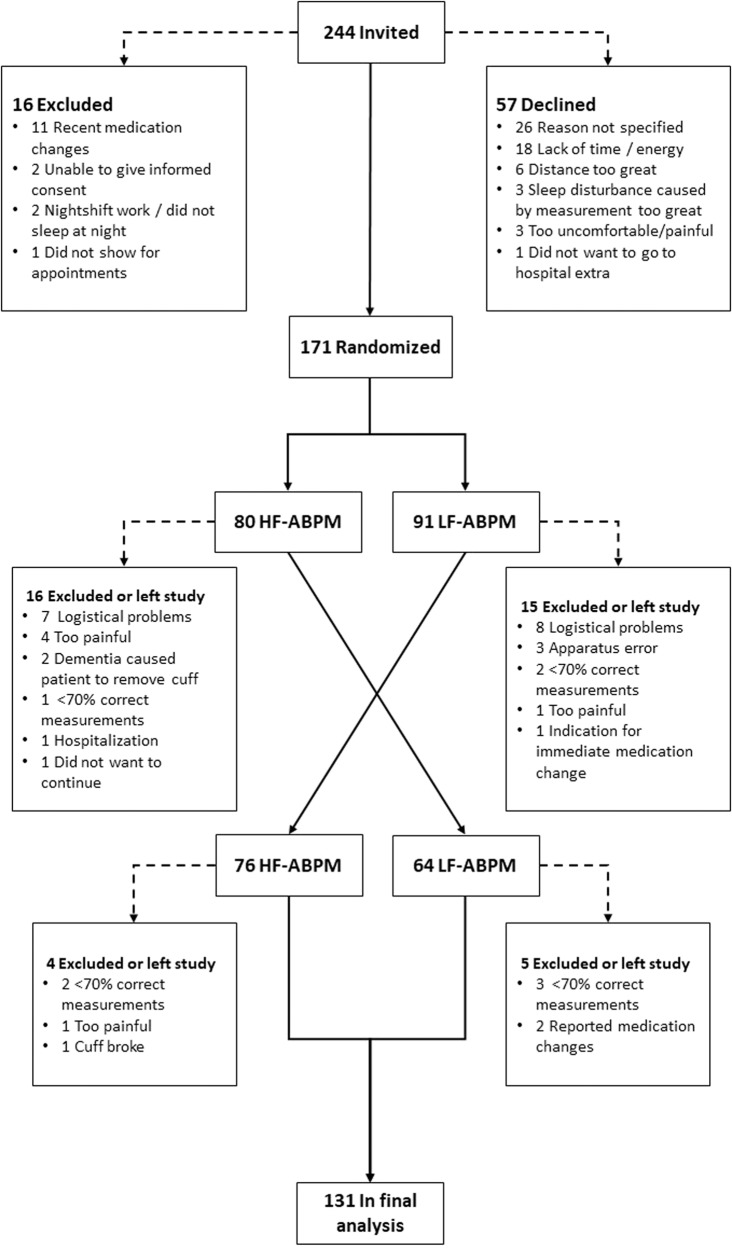
Flowchart of inclusion. HF-ABPM High frequency ambulatory blood pressure monitoring, LF-ABPM Low frequency ambulatory blood pressure monitoring.

A total of 171 patients were randomized. However, 40 (23.4%) did not complete the study or were excluded because of reasons outlined in Fig. 1. These 40 patients did not differ from the 131 patients included in the final analysis regarding gender, age, smoking habits, or BP. However, they did have a significantly larger body mass index of 30.2 kg/m^2^. “Logistical problems” in Fig. 1 mainly refers to a situation when patients were unable to complete both ABPMs before the next clinical outpatient appointment, which would result in medication change between the two ABPMs.

Baseline characteristics of the 131 patients finalizing both ABPMs are shown as total study population and according to measurement order (HF-ABPM first and LF-APBM first) in Table 1. Various other ABPM details, such as total and successful number of measurements, are presented in Table 2.

**Table 1:** Basic characteristics of the included patients.

	All patients	HF first	LF first
No. patients	131	59	72
Female	62 (47.3)	28 (47.5)	34 (47.2)
Age (years)	58.2 ± 14.2	59.4 ± 13.5	57.2 ± 14.7
Smokers	82 (62.6)	44 (74.6)	38 (52.8)
BMI (kg/m^2^)	27.8 ± 5.9	27.8 ± 5.7	27.8 ± 6.0
CKD	35 (26.9)	18 (30.5)	17 (23.9)
Ischemic heart disease	18 (13.7)	6 (10.2)	12 (16.7)
Diabetes	19 (14.5)	8 (13.6)	11 (15.3)
Normotension/controlled hypertension	32 (24.4)	11 (18.6)	21 (29.2)
Mild hypertension	70 (53.4)	34 (57.6)	36 (50.0)
Moderate/severe hypertension	29 (22.1)	14 (23.7)	15 (20.8)
No. of blood pressure medications	2.6 ± 1.8	2.7 ± 1.9	2.6 ± 1.8

Data are presented as mean ± SD or median (interquartile range) for continuous variables and *n* (%) for categorical variables. Hypertension categories were assigned based on the patients first ABPM. Normotension/controlled hypertension was defined as 24 h SBP/DBP < 130/80 mmHg, mild hypertension as 24 h SBP 130–149 mmHg and moderate/severe hypertension as 24 h SBP ≥ 150 mmHg. *HF* High frequency, *LF* Low frequency, *BMI* Body mass index, *CKD* Chronic kidney disease, *eGFR* Estimated glomerular filtration rate, *UACR* Urine albumin creatinine ratio, *SBP* Systolic blood pressure.

**Table 2:** Ambulatory blood pressure monitoring characteristics of the included patients.

	All patients	HF-ABPM first	LF-ABPM first
First ABPM, *n* (%)	45 (34.3)	22 (37.3)	23 (31.9)
Days between ABPMs	5 (5;6)	5 (4;6)	5 (5;7)
HF-ABPM total no. of measurements	72.6 ± 6.7	73.4 ± 6.1	71.9 ± 7.1
LF-ABPM total no. of measurements	27.3 ± 2.5	27.5 ± 2.3	27.0 ± 2.7
Total no. of correct measurements HF-ABPM	62 (58;64)	61 (58;64)	63 (59;64)
Total no. of correct measurements LF-ABPM	23 (22;24)	23 (22;24)	24 (22;24)
% Correct measurements HF-ABPM	96.9 (90.6;100.0)	95.3 (90.6;100.0)	97.7 (91.4;100.0)
% Correct measurements LF-ABPM	95.8 (91.7;100.0)	95.8 (91.7;100.0)	100.0 (91.7;100.0)

Data are presented as mean ± SD or median (interquartile range) for continuous variables and number (%) for categorical variables. Percentage correct measurements are the total number of correct measurements divided by the maximal number of possible correct measurements (24 for LF-ABPM and 64 for HF-ABPM). *ABPM* Ambulatory blood pressure monitoring, *HF* High frequency, *LF* Low frequency.

### 24 h blood pressure differences and agreement

**Fig. 2:**
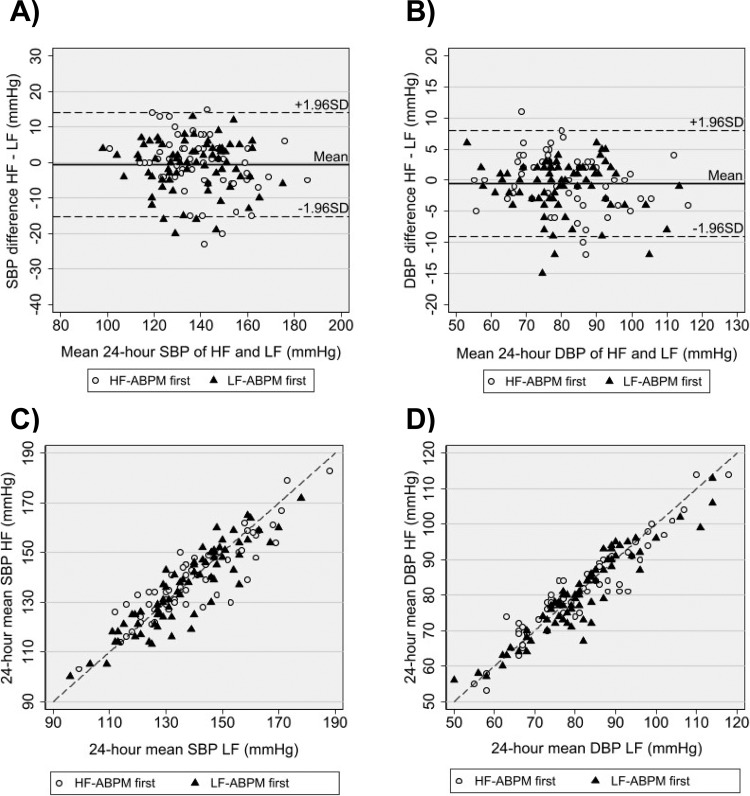
24 h blood pressures. Bland-Altman plots (**A**, **B**) and scatterplots (**C**, **D**) of 24 h systolic (SBP) and diastolic BP (DBP) means for high-frequency ABPM (HF) and low-frequency ABPM (LF). In (**A**, **B**) is the mean difference represented by the solid line and 95% limits of agreement (±1.96 SD) by the dashed lines. In (**C**, **D**) the line of equality is represented by the dashed line.

There were no significant differences in 24 h BP means between HF-ABPM and LF-ABPM and a high level of agreement between the two measurement protocols. As shown in Table 3, 24 h mean SBP was 137.5 mmHg (95% CI, 134.8;140.2) and 138.2 mmHg (95% CI, 135.2;141.1) for HF-ABPM and LF-ABPM respectively resulting in a mean difference (HF-ABPM – LF-ABPM) of −0.7 mmHg (95% CI, −2.0;0.6). The 95% LoA for 24 h SBP were −15.3 mmHg and +14.0 mmHg (Fig. 2A). The mean DBP difference was −0.6 mmHg with 95% LoA between −9.1 mmHg and +8.0 mmHg (Fig. 2B). The scatter plots shown in Fig. 2C, D display a high level of agreement between 24 h SBP and DBP values obtained with the two measurement frequencies. The SBP difference between HF-ABPM and LF-ABPM did not seem to change when the number of succesful measurements during LF-ABPM was less than 24 (Supplementary Fig. 1).

**Table 3:** Blood pressure values and coefficients of variance during 24-hour ambulatory blood pressure monitoring.

Period	HF-ABPM	LF-ABPM	Difference	*P*-value
**24** **h**				
SBP	137.5 (134.8;140.2)	138.2 (135.2;141.1)	−0.7 (−2.0;0.6)	0.312
DBP	80.4 (78.3;82.5)	81.0 (78.7;83.2)	−0.6 (−1.3;0.2)	0.146
SBP CV	11.3 (10.8;11.8)	11.5 (10.8;12.1)	−0.1 (−0.6;0.4)	0.607
DBP CV	13.3 (12.8;13.8)	13.3 (12.7;14.0)	−0.1 (−0.6;0.5)	0.798
**Daytime**				
SBP	142.1 (139.2;145.0)	143.1 (140.1;146.2)	−1.0 (−2.3;0.4)	0.156
DBP	84.2 (82.0;86.5)	85.0 (82.6;87.4)	−0.7 (−1.6;0.1)	0.070
SBP CV	9.7 (9.2;10.1)	9.7 (9.0;10.3)	0.0 (−0.5,0.5)	0.939
DBP CV	10.8 (10.3;11.3)	10.5 (9.9;11.1)	0.3 (−0.3;0.9)	0.326
**Nighttime**				
SBP	127.8 (124.9;130.8)	129.4 (126.1;132.7)	−1.6 (−3.4;0.2)	0.073
DBP	72.6 (70.7;74.6)	73.8 (71.7;76.0)	−1.2 (−2.2;−0.1)	0.026
SBP CV	9.3 (8.8;9.8)	9.2 (8.6;9.8)	0.1 (−0.5;0.7)	0.702
DBP CV	11.7 (11.0;12.3)	11.6 (10.9;12.2)	0.1 (−0.6;0.8)	0.805

Data are mean with 95% confidence intervals. The difference is calculated as HF-ABPM – LF-ABPM. *HF-ABPM* High frequency ambulatory blood pressure monitoring, *LF-ABPM* Low frequency ambulatory blood pressure monitoring, *SBP* Systolic blood pressure, *DBP* Diastolic blood pressure, *CV* Coefficient of variation.

### Daytime and nighttime blood pressure differences and agreement

**Fig. 3:**
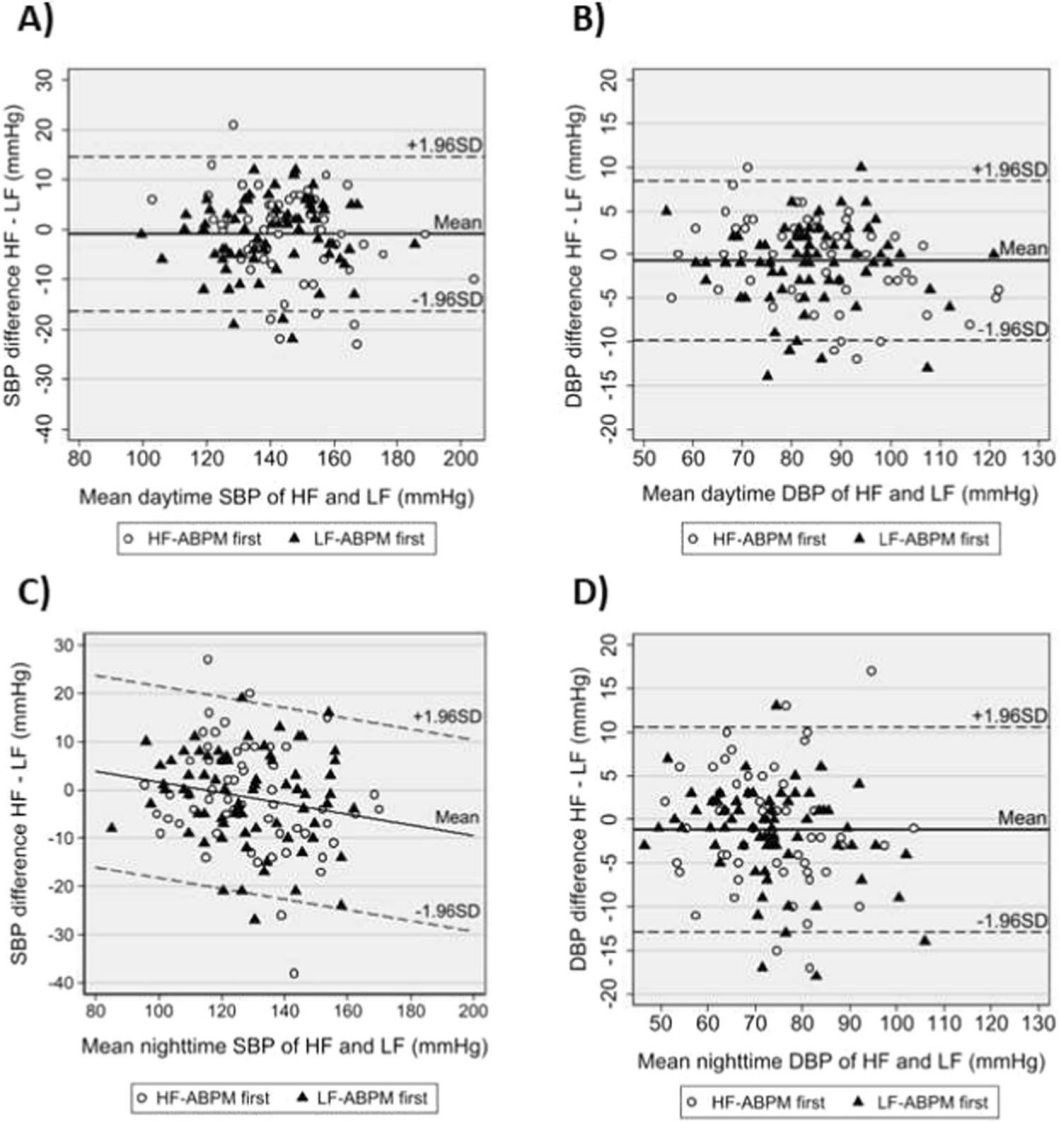
Day and nighttime blood pressures. Bland-Altman plots for daytime SBP (**A**), daytime DBP (**B**) nighttime SBP (**C**) and nighttime DBP (**D**) comparing HF- and LF ABPM. The mean difference is represented by the solid line and 95% limits of agreement (±1.96 SD) by the dashed lines. Only nighttime SBP demonstrated a significant change in the difference between HF- and LF-ABPM with changes in mean blood pressure.

Daytime and nighttime averages were similar for the two measurement frequencies. Table 3 presents the means and differences with 95% CI for 24 h-, daytime-, and nighttime ABPM values. Table 3 also presents CV for 24 h, daytime- and nighttime BP. Of particular interest, no differences in CV between HF-ABPM and LF-ABPM were found. Reducing the number of succesful measurements seemed to slightly increase the CV for both HF-ABPM and LF-ABPM (Supplementary Figs. 2, 3). The agreement between the two methods for daytime and nighttime values are presented as Bland-Altman plots in Fig. 3A–D. The mean differences were −1.0 mmHg (95% LoA, −16.4 mmHg to +14.5 mmHg) for daytime SBP (Fig. 3A), −0.7 mmHg (95% LoA, −9.9 mmHg to +8.4 mmHg) for daytime DBP (Fig. 3B), and −1.2 mmHg (95% LoA, −12.9 mmHg to +10.6 mmHg) for nighttime DBP (Fig. 3D). For nighttime SBP there was a statistically significant negative linear correlation between increasing BP values and the difference between HF-ABPM and LF-ABPM as shown in Fig. 3C. For nighttime SBP the correlation coefficient was −0.11 mmHg (95% CI −0.21;−0.01), meaning for every 10 mmHg increase in mean SBP the difference between HF-ABPM and LF-ABPM increased with −1.1 mmHg (the difference was 0 mmHg at mean nighttime SBP of 120 mmHg).

We found that 49.6% of patients had a concordant dipping status between LF-ABPM and HF-ABPM (Supplementary Table 1). If grouping patients only by dipping (“dipper” and “extreme-dippers” combined) or non-dipping (“non-dipper” and “reverse-dipper” combined) we found 74% to have a concordant dipping status. When examining each sex independently, there were no difference in any BP parameters between HF-ABPM and LF-ABPM.

### First time ABPM, hypertension categories, and order of measurement

Patients who underwent ABPM for the first time in the present study (*n* = 45) did not differ from those previously exposed to ABPM. They had a mean 24 h SBP of 136.3 mmHg (95% CI, 132.5;140.2) during HF-ABPM and 137.0 mmHg (95% CI, 133.0;141.1) during LF-ABPM, resulting in a difference of −0.7 mmHg (95% CI, −3.1;1.7).

For patients with moderate/severe hypertension LF-ABPM measured all but daytime SBP and nighttime DBP slightly higher compared to HF-ABPM (Supplementary Table 2). For mildly hypertensive and normotensive/controlled patients there were no BP differences between HF-ABPM and LF-ABPM.

There were no differences in neither 24 h, daytime, and nighttime BP values between those randomized to HF-ABPM first and those randomized to LF-ABPM first.

### Questionnaire on pain and sleep

Most patients rated ABPM-related pain low on a VAS. However, patients reported HF-ABPM to be slightly more painful compared to LF-ABPM (Table 4). Patients reported pain related to daytime cuff-inflations to be higher than nighttime cuff-inflation for both LF-ABPM and HF-ABPM. Five patients left the study because they found HF-ABPM “too painful to continue”, for LF-ABPM this was only true for one patient, however, this difference was not statistically significant (*P* = 0.1). Significantly more patients reported “no symptoms” at the cuff-site during LF-ABPM compared to HF-ABPM (Table 4). Patients who underwent their first ABPM (*n* = 45) tended to rate HF-ABPM as more painful compared to LF-ABPM, but this did not reach statistical significance (daytime *P* = 0.097 and nighttime *P* = 0.056). Sleep duration and sleep interruption did not differ between the two measurement protocols (Table 4).

**Table 4:** Questionnaire data.

	HF-ABPM	LF-ABPM	*P*-value
**Pain (*n*** = 122)			
VAS daytime	1.5 (0.6;3.0)	1.3 (0.6;2.9)	0.029
VAS nighttime	1.3 (0.4;3.4)	0.9 (0.4;2.0)	0.005
**Sleep (*n*** = 131)			
Hours of sleep	8.1 ± 1.2	8.2 ± 1.1	0.117
Interrupted sleep^†^	55 (44.7)	46 (37.4)	0.072
**Symptoms after removal of cuff (*n*** = 131)			
No symptoms	77 (58.8)	91 (69.5)	0.016
Very red skin	14 (10.7)	8 (6.1)	0.083
Bruises	13 (9.9)	8 (6.1)	0.166
Soreness	18 (12.2)	17 (13.0)	0.808
Other	19 (16.8)	16 (11.5)	0.549

Data are presented as mean ± SD or median (interquartile range) for continuous variables and number (%) for categorical variables. *HF-ABPM* High frequency ambulatory blood pressure monitoring, *LF-ABPM* Low frequency ambulatory blood pressure monitoring, *VAS* Visual analogue scale (0–10), *IQR* Interquartile range. ^†^*n* = 121.

## Discussion

We here present the first randomized study comparing 24 h ABPM with hourly measurements to 24 h ABPM using the currently recommended frequencies of 2–3 measurements per hour in a patient-population covering a broad range of BP levels. We found a good agreement between HF-ABPM and LF-ABPM for both SBP and DBP values. In addition, LF-ABPM was rated slightly less painful during cuff inflation. Our data support that one measurement every hour is sufficient to achieve correct values for BP as well as for BP variability.

### Low vs. high measurement frequency

The present findings are in accordance with two smaller studies, which compared hourly measurements with 4 measurements per hour without detecting any difference in BP values [6, 7]. Our previous non-randomized study indicated SBP to increase during HF-ABPM in moderate to severe hypertensive patients when compared to LF-ABPM [19]. We could not reproduce this finding using a more rigorous study design; rather HF-ABPM nighttime SBP seems slightly lower with increasing BP levels compared to LF-ABPM. However, since this effect was only clinically significant for nighttime BP levels well above the current definition of hypertension, the use of LF-ABPM will not lead to misclassification of any hypertensive patients as normotensive.

The most utilized method for investigating the influence of measurement frequencies is post-hoc random removal of individual measurements after performing a single ABPM, to simulate a lower measurement frequency [8–15]. Using this approach, two studies have compared intraarterial measurements to ABPM and found that hourly measurements constituted the lowest possible frequency at which the 24 h BP estimate remained accurate [8, 15]. Other studies, using only non-invasive ABPM, found that hourly or fewer measurements were comparable to ABPMs following current recommendations [9–12, 14]. However, it is important to stress that the influence of cuff inflation frequency on patient BP and experience of the ABPM cannot be evaluated using the post-hoc random removal methodology.

A possible concern using LF-ABPM could be that failed measurements might influence BP estimation to a larger degree than for HF-ABPM. Our study was not designed to answer this concern, but we found no indication of this as SBP difference between HF-ABPM and LF-ABPM did not seem to change with fewer successful measurements during LF-ABPM. However, since only 12 patients had less than 20 successful measurements, this finding is uncertain. Yang et al. found as few as 8 valid daytime readings and 4 valid nighttime readings were enough for results to be comparable to what was attained using current guideline recommended frequencies [10], supporting our finding that LF-ABPM is feasible for BP estimation, even with some failed measurements.

### Agreement between HF-ABPM and LF-ABPM

Only a few of the studies investigating measurement frequency reported 95% LoA or SD of difference, from which 95% LoA can be calculated [9, 15, 19]. As the current study compared two ABPMs performed on two separate days it is reasonable to compare 95% LoA with other studies investigating reproducibility of ABPM. The reproducibility studies find similar or larger 95% LoA compared to our findings [15, 24–27]. Two recent studies found an LoA interval of ±20 mmHg for 24 h SBP [26, 27]. One used two measurements per hour [27] and the other a measurement frequency similar to our HF-ABPM protocol [26]. In our study, the SBP LoA interval was only ±15 mmHg. Thus, the agreement between HF-ABPM and LF-ABPM in our study is comparable to conducting both ABPMs as HF-ABPM. Agreements concerning dipping status between LF-ABPM and HF-ABPM were similar to another study investigating the reproducibility of dipping status [28].

### Blood pressure variability

It has been argued that an ABPM measurement frequency of once every hour could limit correct assessment of BP variability [9, 13]. Since short-term BP variability is an independent predictor of patients’ cardiovascular risk [29–31], a lower accuracy of this parameter might affect cardiovascular risk estimation. Based on an analysis of BP variability, a previous study concluded that hourly measurements are too inaccurate and the authors therefore recommended no less than two measurements an hour [13]. Our estimate of CV is comparable to other studies with similar populations regarding BP and age [32, 33]. We found no difference in CV between LF-ABPM and HF-ABPM, suggesting that BP variability does not differ substantially between LF-ABPM and HF-ABPM. In theory more measurements could result in a lower variation, however BP has an innate circadian variation which limits how low the variation can be. This is exemplified by a study by Di Rienzo et al. in which intraarterial mean pressure was assessed with thousands of measurements per patient reaching a CV similar to ours [8]. LF-ABPM seems to capture the innate circadian variation of BP sufficiently, resulting in no CV difference between LF-ABPM and HF-ABPM.

### Sleep quality and pain

Some patients report pain from cuff inflations as a major complaint in relation to ABPM [3, 34]. In the present study, patients generally rated pain as “low” (50% of patients rated pain lower than 1.5/10) for both frequencies. A few did, however, rate the pain very high with two patients rating it 10/10 (“worst possible pain”) for both frequencies. HF-ABPM was overall reported more painful compared to LF-ABPM. However, while the difference was statistically significant, the clinical relevance needs to be clarified, but, ideally, we aim for ABPM regimens causing the lowest amount of pain.

Earlier studies have emphasized that interrupted sleep and a subsequent possible increase in nighttime BP as limitations of ABPM [3–5, 35], but a recent, larger study found only 21% of patients undergoing ABPM had mild sleep disturbances and nighttime BP did not increase [33]. Despite of a tendency toward more interrupted sleep, we did not find evidence that cuff-inflations twice every hour affects nighttime BP more than hourly cuff inflations.

### Limitations and strengths

A possible limitation of all hospital-based hypertension studies is external validity and whether findings can be extrapolated to other settings. Patients referred to a specialized hypertension clinic more frequently have treatment-resistant hypertension or more advanced end-organ damage compared to patients treated in primary care clinics.

LF-ABPM is the standard practice in our clinic and several patients from our cohort had undergone one or more ABPMs before inclusion. Patients who previously underwent LF-ABPM, and found it painful or stressful, may decline participation in a study involving several ABPMs. However, when comparing patients who underwent ABPM for the first time with the total study population, we found no difference in 24 h BP or their rating of pain and sleep disturbances. We consider the risk of selection bias to be minimal based on the similarity between first-time ABPM patients and patients with prior ABPM experience. The low number of patients who reported “too uncomfortable/painful” as the reason to decline participation in the study further supports this.

## Conclusion

This is the first randomized study to investigate whether ABPM using hourly measurements may be an alternative to current guideline-recommended measurement frequencies. We found good agreement and no clinically significant BP differences between the two measurement frequencies. In addition, there was a modest reduction in reported pain during LF-ABPM compared to HF-ABPM. ABPM using hourly measurements might increase patient acceptance of repeated ABPMs without reducing data quality and thereby potentially increase BP control. Future research should focus on investigating the usefulness of LF-ABPM in predicting cardiovascular risk compared to HF-ABPM.

## Summary

### What is known about the topic

- Current guidelines recommend 2–3 measurements per hour during ambulatory blood pressure monitoring (ABPM) but provide no clear evidence for this.
- Previous studies have tried to mimic a lower measurement frequency by random removal of data from ABPM using 2–3 recordings per hour.
- A direct comparison of a high *vs* a low frequency cuff inflation protocol during 24 h ABPM has never been performed in a relevant population of hypertensive patients.

### What this study adds

- First randomized trial to actually compare hourly measurements with current guideline recommendations.
- There are no clinically relevant differences in neither blood pressure nor blood pressure variation between the two measurement frequencies.
- ABPM related pain was significantly less both during daytime and nighttime when using hourly measurements.
- Our data suggest that ABPM using hourly measurements is an alternative to current guideline recommendations.

### Supplementary information

Supplementary tables and figures

## Data Availability

The raw data that support the findings of this study are currently safely stored at our research facility. It contains patient specific data and is therefore not publicly available. This is in agreement with the General Data Protection Regulation of the European Union. Data can be made available upon reasonable request.
