# Supplementary material for: High versus low measurement frequency during 24-h ambulatory blood pressure monitoring - a randomized crossover study
Source: J Hum Hypertens. 2023 Oct 11;38(2):146–54. doi: 10.1038/s41371-023-00868-0 (PMC10844074; doi:10.1038/s41371-023-00868-0)
Supplement: Supplementary file 1 — Supplementary tables and figures [file 41371_2023_868_MOESM1_ESM.pdf]

Supplemental Materials

Supplemental Tables:

Supplemental table 1 – Dipping status between HF-ABPM and LF-ABPM

HF-ABPM / LF-ABPM	Reverse dipper	Non-dipper	Dipper	Extreme Dipper	Total
**Reverse dipper**	7 (5.3)	11 (8.4)	0 (0.0)	0 (0.0)	18 (13.7)
**Non-dipper**	7 (5.3)	22 (16.8)	18 (13.7)	1 (0.8)	46 (35.1)
**Dipper**	2 (1.5)	15 (11.5)	28 (21.4)	7 (5.3)	52 (39.7)
**Extreme dipper**	0 (0.0)	0 (0.0)	7 (5.3)	8 (6.1)	15 (11.5)
**Total**	16 (12.2)	48 (36.6)	51 (38.9)	16 (12.2)	131 (100)

Data are presented as n and % of 131 in parentheses. Reverse dipping was defined as night/day SBP ratio >1.0. Non-dipping was defined as night/day SBP ratio between 0.9-1.0. Dipping was defined as night/day SBP ratio between 0.8-0.9. Extreme dipping was defined as night/day SBP ratio <0.8. HF-ABPM, high frequency ambulatory blood pressure monitoring; LF-ABPM, low frequency ambulatory blood pressure monitoring; SBP, systolic blood pressure.

Supplemental table 2 – Systolic blood pressure and diastolic blood pressure differences between HF-ABPM and LF-ABPM for hypertension categories.

	**Normotensive/controlled hypertension n=32**	**Mild hypertension n=70**	**Moderate/severe hypertension n=29**
**24-hour SBP difference**	0.9 (-0.9;2.8)	-0.4 (-2.4;1.6)	-3.0* (-5.7;-0.3)
**24-hour DBP difference**	0.5 (-0.7;1.7)	-0.6 (-1.7;0.5)	-1.6* (-3.2;0.0)
**Day SBP difference**	0.2 (-2.1;2.5)	-0.7 (-2.7;1.3)	-2.9 (-6.0;0.2)
**Day DBP difference**	0.1 (-1.2;1.4)	-0.6 (-1.8;0.7)	-2.1* (-3.8;-0.4)
**Night SBP difference**	0.3 (-2.4;2.9)	-1.2 (-3.9;1.5)	-4.7* (-8.5;-0.9)
**Night DBP difference**	0.1 (-1.6;1.8)	-1.5 (-3.0;0.0)	-1.9 (-4.4;0.7)

Data are presented as mean (95% CI). Differences are calculated as HF-ABPM – LF-ABPM. Hypertension categories were assigned based on the patients first ABPM. Normotension/controlled hypertension was defined as 24-hour SBP/DBP <130/80 mmHg, mild hypertension as 24-hour SBP 130-149 mmHg and moderate/severe hypertension as 24-hour SBP ≥150 mmHg. SBP, systolic blood pressure; DBP, diastolic blood pressure; HF-ABPM, high frequency ambulatory blood pressure monitoring; LF-ABPM, low frequency ambulatory blood pressure monitoring. * $P < 0.05$ paired t-test comparing LF-ABPM and HF-ABPM means.

Supplemental figures:

Supplemental Figure 1

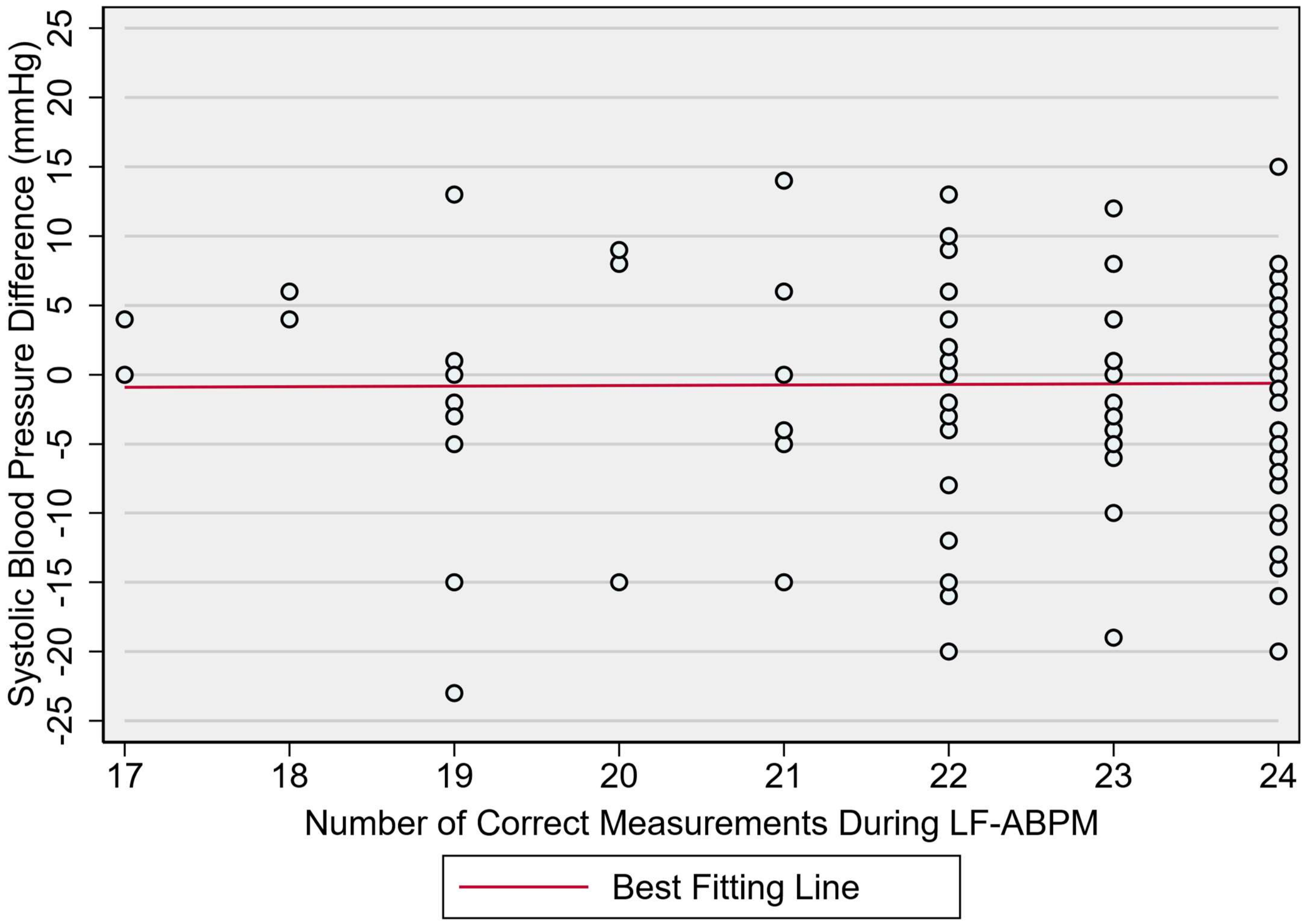

Figure legend: Scatterplot of 24-hour SBP difference and number of correct measurements during LF-ABPM. 24-hour SBP difference calculated as HF-ABPM – LF-ABPM. Solid line represents the best fitting line of a linear regression. LF-ABPM, low frequency ambulatory blood pressure monitoring; HF-ABPM, high frequency blood pressure monitoring; SBP, systolic blood pressure.

Supplemental Figure 2

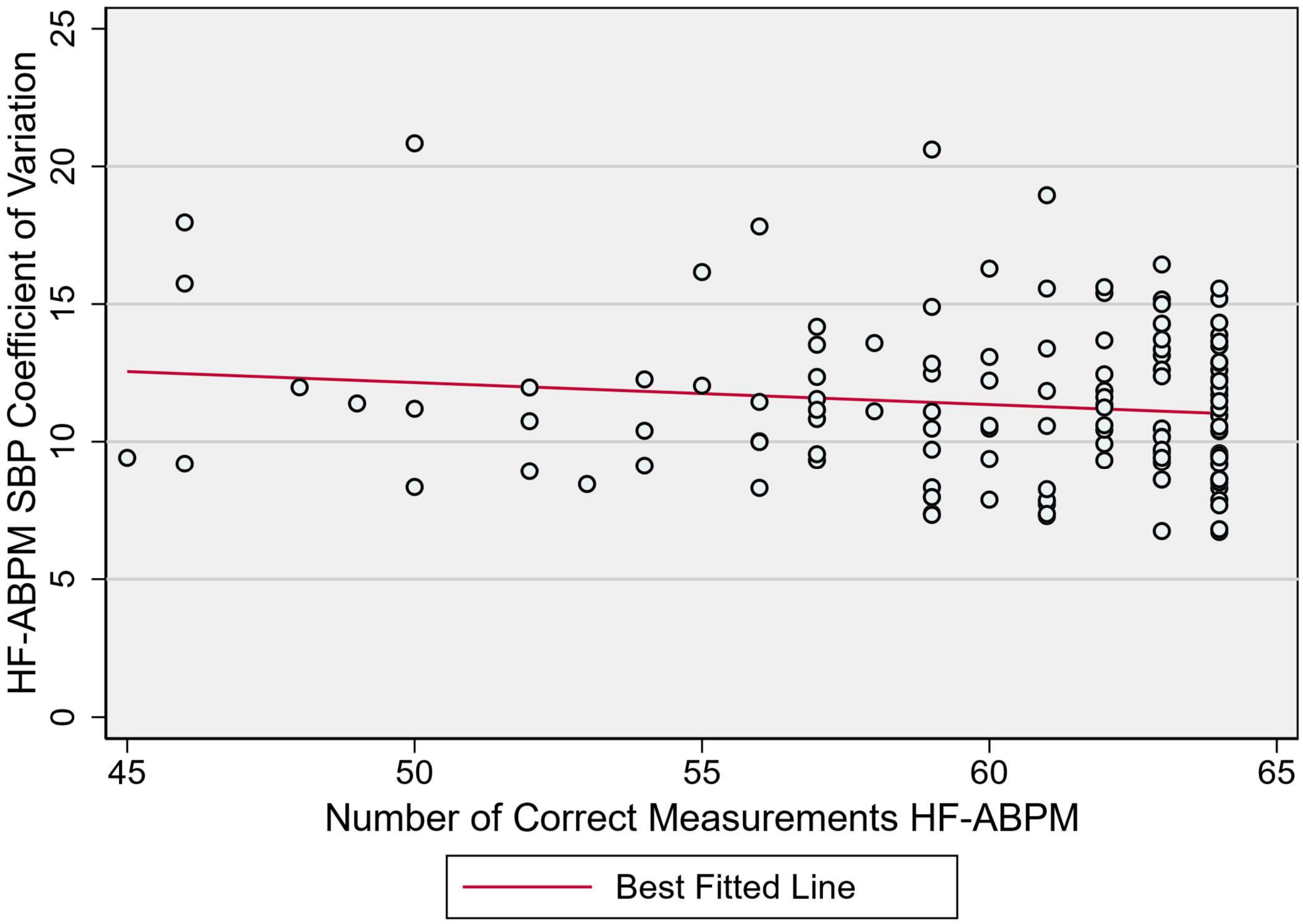

Figure legend: Scatterplot of HF-ABPM SBP coefficient of variation and number of correct measurements during HF-ABPM. Solid line represents the best fitting line of a linear regression. HF-ABPM, high frequency blood pressure monitoring; SBP, systolic blood pressure.

Supplemental Figure 3

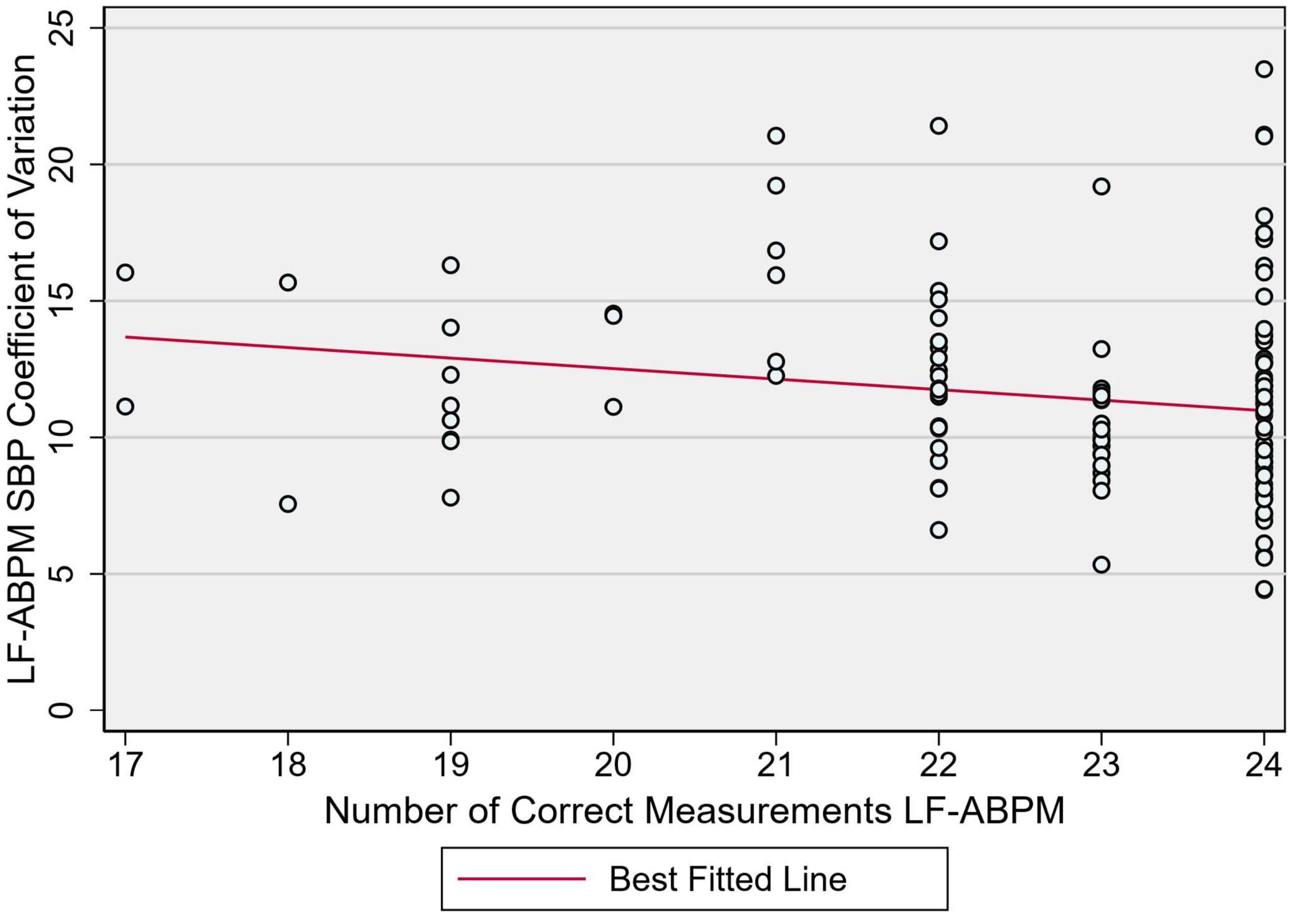

Figure legend: Scatterplot of LF-ABPM SBP coefficient of variation and number of correct measurements during LF-ABPM. Solid line represents the best fitting line of a linear regression. LF-ABPM, low frequency blood pressure monitoring; SBP, systolic blood pressure.
